# *Mgll* Knockout Mouse Resistance to Diet-Induced Dysmetabolism Is Associated with Altered Gut Microbiota

**DOI:** 10.3390/cells9122705

**Published:** 2020-12-17

**Authors:** Niokhor Dione, Sébastien Lacroix, Ulrike Taschler, Thomas Deschênes, Armita Abolghasemi, Nadine Leblanc, Vincenzo Di Marzo, Cristoforo Silvestri

**Affiliations:** 1 Département de Médecine, Université Laval, Québec, QC G1V 0A6, Canada; ndione@stanford.edu (N.D.); armita.abolghasemi.1@ulaval.ca (A.A.); vincenzo.dimarzo@criucpq.ulaval.ca (V.D.M.); 2 Canada Excellence Research Chair on the Microbiome-Endocannabinoidome Axis in metabolic Health, Québec, QC G1V 4G5, Canada; sebastien.lacroix.8@ulaval.ca (S.L.); thomas.deschenes.1@ulaval.ca (T.D.); Nadine.Leblanc@fsaa.ulaval.ca (N.L.); 3 Institut Universitaire de Cardiologie et de Pneumologie de Québec, Université Laval, Québec, QC G1V 4G5, Canada; 4 Institut sur la Nutrition et les Aliments Fonctionnels, Université Laval, Québec, QC G1V 0A6, Canada; 5 Faculté des Sciences de L’agriculture et de L’alimentation, Université Laval, Québec, QC G1V 0A6, Canada; 6 Institute of Molecular Biosciences, University of Graz, 8010 Graz, Austria; ulrike.taschler@uni-graz.at; 7 Institute of Biomolecular Chemistry, National Research Council, 80078 Pozzuoli, NA, Italy

**Keywords:** monoglyceride lipase, endocannabinoids, 2-arachidonoyl glycerol, monoacylglycerols, microbiota

## Abstract

Monoglyceride lipase (MGLL) regulates metabolism by catabolizing monoacylglycerols (MAGs), including the endocannabinoid 2-arachidonoyl glycerol (2-AG) and some of its bioactive congeners, to the corresponding free fatty acids. *Mgll* knockout mice (*Mgll*^−/−^) exhibit elevated tissue levels of MAGs in association with resistance to the metabolic and cardiovascular perturbations induced by a high fat diet (HFD). The gut microbiome and its metabolic function are disrupted in obesity in a manner modulated by 2-arachidonoyl glycerol (2-AG’s) main receptors, the cannabinoid CB1 receptors. We therefore hypothesized that *Mgll*^−/−^ mice have an altered microbiome, that responds differently to diet-induced obesity from that of wild-type (WT) mice. We subjected mice to HFD and assessed changes in the microbiomes after 8 and 22 weeks. As expected, *Mgll*^−/−^ mice showed decreased adiposity, improved insulin sensitivity, and altered circulating incretin/adipokine levels in response to HFD. *Mgll*^−/−^ mice on a chow diet exhibited significantly higher levels of *Hydrogenoanaerobacterium*, *Roseburia*, and *Ruminococcus* than WT mice. The relative abundance of the *Lactobacillaceae* and *Coriobacteriaceae* and of the *Lactobacillus*, *Enterorhabdus*, *Clostridium*_*XlVa*, and *Falsiporphyromonas* genera was significantly altered by HFD in WT but not *Mgll*^−/−^ mice. Differently abundant families were also associated with changes in circulating adipokine and incretin levels in HFD-fed mice. Some gut microbiota family alterations could be reproduced by supplementing 2-AG or MAGs in culturomics experiments carried out with WT mouse fecal samples. We suggest that the altered microbiome of *Mgll*^−/−^ mice contributes to their obesity resistant phenotype, and results in part from increased levels of 2-AG and MAGs.

## 1. Introduction

The gut microbiota has been the subject of numerous studies in recent years resulting in important discoveries highlighting the importance of the relationship between the host and its enteric microorganisms, collectively termed the microbiome. It is involved in many metabolic and chronic pathologies either via direct interactions with the intestinal wall or indirectly through production of bacterial metabolites or the modification of host-derived factors [1,2].

The two endocannabinoids, i.e., 2-arachidonoyl glycerol (2-AG) and *N*-arachidonoylethanolamine (anandamide; AEA), their biochemically related congeners (i.e., the *N*-acylamines (e.g., *N*-acylethanolamines, *N*-acylethaminoacids etc.) and monoacylglycerols; MAGs), metabolizing enzymes and receptors together form the endocannabinoidome (eCBome) [3]. While it is well accepted that 2-AG and AEA, primarily signaling through the cannabinoid receptors (CB1 and CB2) both within the brain and peripheral tissues, regulate whole-body metabolism [4], increasing evidence supports the notion that the eCBome in general is involved in regulating metabolism, with effects that often counter those of the classic endocannabinoids [4,5,6].

Manipulation of the eCBome, either genetically [7,8] or pharmacologically [9,10,11,12], impacts upon energy metabolism in association with changes in the gut bacterial community, the metabolic effects of which were partially replicated via fecal microbiota transplantation to naïve mice [8,11]. Conversely, modification of the gut microbiota community with prebiotics, probiotics, antibiotics, vitamins or diet, or its complete removal resulting in germ-free mice, modulates the eCBome lipid mediator and gene expression levels in intestinal and adipose tissues [13,14,15,16,17,18]. There is thus evidence of bidirectional interactions between the gut microbiome and the eCBome leading to the hypothesis of the existence of a microbiome-eCBome axis that is responsive to external factors and is critical for the regulation of host metabolic health at many levels [4,6,19].

Several genetic mouse models in which the eCBome has been altered through the manipulation of enzymes or receptors have been produced, many of which may be useful for gaining a further understanding of the microbiome-eCBome axis. Monoglyceride lipase (MGLL) knockout mice (*Mgll*^−/−^) is one such model [20]. The enzyme encoded by this ubiquitously expressed gene is considered to be the rate limiting enzyme for the metabolism of MAGs to the corresponding free fatty acids and glycerol, and thus is critical for lipid and energy metabolism, but is also at the crossroads of a plethora of processes such as cognitive functions, pain sensations, and inflammation [21,22]. MGLL is a critical member of the eCBome as it catalyzes the hydrolysis of the endocannabinoid 2-AG and other MAGs and thus intervenes also in neutral glycerolipid metabolism [22]. In some organs, such as the brain, MGLL is also involved in an alternative pathway for the production of arachidonic acid (AA) serving as a biosynthetic precursor of prostanoids and other eicosanoids [22]. As an endocannabinoid, 2-AG can activate both CB1 and CB2 receptors, which have been implicated in the regulation of food intake, energy metabolism, and inflammation [3,4,23]. Increased 2-AG levels and/or CB1 hyperactivity are observed in obese individuals and considered a key contributor to the development and etiopathology of obesity and associated metabolic disturbances [24,25,26,27]. Previous work investigated the impact of MGLL deficiency on the metabolism of mice fed a high-fat diet (HFD) [21,28,29,30]. *Mgll*^−/−^ mice exhibit elevation in 2-AG and other MAGs in brain, liver, and adipose tissue and are protected from diet-induced obesity and metabolic disturbances. In fact, these mice do not exhibit significant hyperglycemia, elevations in plasma, and liver triacylglycerol, have suppressed intestinal lipid absorption following oral challenges compared to their wild-type (WT) littermates, and do not develop glucose intolerance nor insulin resistance following prolonged HFD feeding. The protective effect against insulin resistance appears to be independent of obesity as initial studies on *Mgll*^−/−^ mice found that the mice could gain weight like WT mice but maintained insulin sensitivity to a greater extent [20]. Central and intestinal CB1 desensitization due to 2-AG elevation was observed in *Mgll*^−/−^ mice as a homeostatic mechanism to prevent CB1 hyperactivity [20,22,29]. Similar compensatory mechanisms are also observed in chronic cannabis users [31]. However, the observed metabolic benefits may also arise from the elevation of other eCBome MAGs and their subsequent activation of metabolically favorable signaling pathways (e.g., via GPR119 or TRPV1). *Mgll*^−/−^ mice are therefore an interesting model in which to study the effects of overactivated/desensitized CB1, and/or enhanced GPR119 and TRPV1 [32], activity on the gut microbiome, and thus identify bacterial species directly impacted by these receptors and their role in metabolism.

The main objective of this study was to determine if MGLL modulates the gut microbiome of mice and its response to a HFD in order to gain an understanding of the mechanisms by which *Mgll*^−/−^ mice are resistant to diet induced obesity and related metabolic perturbations.

## 2. Materials and Methods

### 2.1. Mice, Diets, and Stool Sample Collection

*Mgll*^−/−^ mice were generated as described previously [20]. Mice were backcrossed onto C57BL/6J for at least 10 generations. Mice were bred and maintained on a regular light dark cycle (14 h light, 10 h dark) at 22 ± 1 °C in a barrier facility in specific pathogen free quality. Animals had ad libitum access to chow diet (12 kJ% fat, V1126, Ssniff Spezialdiaeten GmbH, Soest, Germany) or were fed HFD (45kJ% fat, E15744, Ssniff Spezialdiaeten GmbH, Soest, Germany). HFD-treatment started at 6 weeks of age for 22 weeks. Body mass composition was determined by TD-NMR miniSpec Live Mice Analyzer (Bruker Optics, Billerica, MA, USA). Stool samples were collected three times from all mice, at 6 weeks of age (6 weeks old), after 8 weeks of HFD (14 weeks old) and after 22 weeks of HFD (28 weeks old). All the samples were stored at −80 °C until further use. All animal experiments were approved by the Austrian Federal Ministry for Science, Research, and Economy (protocol number BMBWF-66.007/0030-V/3b/2018) and the ethics committee of the University of Graz, and were conducted in compliance with the council of Europe Convention (ETS 123).

### 2.2. Glucose Tolerance Test and Plasma Cytokine Analysis

For intraperitoneal glucose tolerance tests (ipGTT), mice were fasted 6 h (7 a.m.–1 p.m.) and then injected intraperitoneally with a 20% glucose solution (1.5 g/kg). Blood glucose levels were determined at 0, 15, 30, 60, 120, and 180 min using a Wellion Calla glucometer (MedTrust, Marz, Austria).

For cytokine analysis, all blood samples were collected after an overnight fast (14 h), EDTA plasma was prepared and stored at −80 °C until analysis. A multiplex bead-based immunoassay was used to analyze the diabetes plasma soluble molecules including ghrelin, gastric inhibitory peptide (GIP), glucagon-like peptide-1 (GLP-1), glucagon, insulin, leptin, plasminogen activator inhibitor 1 (PAI-1), and resistin according to the manufacturer’s instruction (Bio-Plex Pro™ mouse diabetes 8-plex assay, Bio-Rad Lab Ltd., Mississauga, ON, Canada) and adiponectin (Bio-Plex Pro mouse diabetes adiponectin assay, Bio-Rad Lab., Inc., Hercules, CA, USA). Plasma levels of all molecules were determined using a Bio-Plex^®^ 200 system with Bio-Plex Manager™ software version 6.0 (Bio-Rad Lab Ltd., Mississauga, ON, Canada).

### 2.3. 16S rDNA Sequencing and Metagenomics Analysis

DNA was extracted using QIAamp PowerFecal DNA kit (Germantown Road Germantown, MD 20874). Sequencing of the V3–V4 regions of rRNA 16S was performed using the following primers 341F (5-CCTACGGGNGGCWGCAG-3) and 805R (5-GACTACHVGGGTATCTAATCC-3) on MiSeq technology (Illumina, Inc, San Diego CA 92121, USA).

Preprocessing of obtained sequences and bacterial taxa assignation was performed according to the Dada2 pipeline (Version 1.10.1) using the Ribosomal Database Project (RDP release 11) reference database [33] (Sequences were filtered to keep only those present in at least five samples. Analyses were then conducted on sequence counts normalized by Cumulative Sum Scaling (CSS) (MetagenomeSeq R package) [34]. CSS divides sequence counts by the median sequence counts of each sample [35]. CSS-normalized counts are thus expressed in relation to the entire bacterial composition of each sample and are viewed as being more appropriate than Total Sum Scaling [36].

### 2.4. Short Chain Fatty Acid Analysis

Short chain fatty acids (SCFAs) were extracted and analyzed with a GC-FID system according to a protocol described by García Villalba et al. (2012) [37]. Briefly, 1 mL of 0.5% phosphoric acid was added per 100 mg of material. Fecal suspensions were homogenized 2 min then centrifuged at 18,000× *g* for 10 min at 4 °C. Supernatants were collected and an equal volume of ethyl acetate, spiked with 4-methylvaleric acid was added. Samples were mixed for 2 min then centrifuged at 18,000× *g* for 5 min at 4 °C. The organic phase was transferred to an autosampler vial for GC-FID analysis.

### 2.5. Targeted Culturomics

Culturomics is a high-throughput method that multiplies culture conditions in order to detect higher bacterial diversity [38]. Briefly, the method consists of diversifying the culture conditions for the same sample (by modifying parameters such as incubation time, temperature, pH, atmosphere, or by adding inhibitory factors such as antibiotics, promoters such as blood, rumen fluid for cultivating minority, and/or fastidious populations) in order to fully explore its microbial composition. Individual culture conditions are detailed in Supplemental Table S1. We used this strategy to target bacteria that could be impacted by 2-AG and monoacylglycerols (MAGs). Stool sample from WT mice was cultured in Complete Hungate Anaerobic tubes (Thermo Fisher Scientific, Waltham, MA, USA) supplemented with either 2-AG (40 µM), AA (40 µM), or total MAGs isolated from the entire intestinal track of a mouse (40 µM). Cultures were incubated at 37 °C in anaerobic condition for 3 days. Then, 2 mL of culture were sampled from each Hungate tube, centrifuged, and the genomic DNA was extracted using the QIAamp PowerFecal DNA kit (Germantown, MD, USA) for 16S sequencing as described above except for the sequencing primers used. Indeed, culturomics samples were sequenced using V2–V3 rRNA 16S specific primers (5-GGCGNACGGGTGAGTAA-3 and 5-WTTACCGCGGCTGCTGG-3, respectively).

### 2.6. Statistical Approach

Data are expressed as mean ± SD (parametric data) or median [Q1–Q3] (nonparametric data). For all boxplots, boxes extend from first to third quartile, vertical lines indicate median and whiskers denote the interquartile range (IQR) from Q1 and Q3 and data outside 1.5 times the IQR are drawn. Two-way ANOVA (taxa abundance ~ Genotype*Diet) followed by Tukey HSD post-hoc were conducted to evaluate the influence of MGLL genotype and HFD on gut microbial taxa. Principal coordinate analyss (PCoA) was performed using Bray–Curtis dissimilarity indexes of beta-diversity and Permutational multivariate analysis of variance (PERMANOVA) were computed to evaluate differences between groups (Vegan R package) [39]. Results were considered statistically significant at *p* < 0.05 or FDR-adjusted *p* < 0.1. Analyses were performed with R software version 3.4.3. [34].

## 3. Results

### 3.1. Mgll^−/−^ Mice Are Resistant to HFD-Induced Obesity and Alterations in Incretin/Adipokine Levels

**Figure 1:**
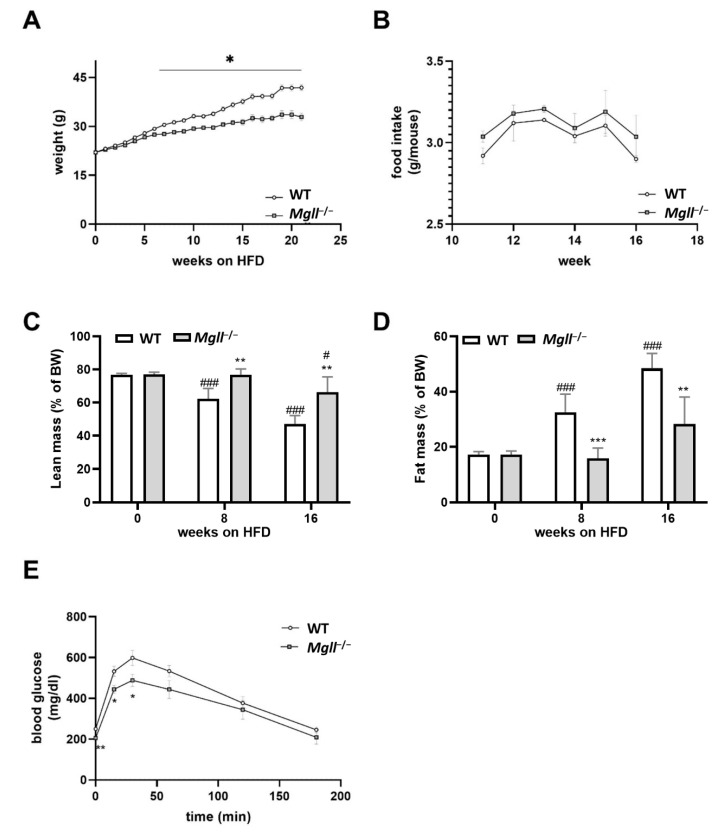
*Mgll*^−/−^ mice are protected from high fat diet- (HFD-)induced obesity and maintain glucose tolerance. (**A**) Longitudinal body weight. (**B**) Weekly mean food intake in HFD-fed WT and *Mgll*^−/−^ mice from week 11 to 16. (**C**) Lean mass and (**D**) fat mass of WT and *Mgll*^−/−^ mice fed HFD analyzed using a TD-NMR miniSpec Live Mice Analyzer at the beginning, and after 8 and 16 weeks of HFD feeding. (**E**) For (intra-peritoneal glucose tolerance test (ipGTT), mice were fasted for 6 h (7 a.m.–1 p.m.) followed by injection of 1.5 g glucose/kg body weight. Blood glucose was determined at indicated time points (male mice, *n* = 6/genotype for all experiments). Data represent mean ± SEM. Statistical significance was determined by Student’s two-tailed *t*-test. * *p* < 0.05, ** *p* < 0.01, *** *p* < 0.001 vs. WT control. # *p* < 0.05, ### *p* < 0.001 vs. week 0 control. BW; body weight.

First, we confirmed the resistance of *Mgll*^−/−^ mice to high fat diet- (HFD-)induced obesity reported by others [28,30,40]. We observed no weight or body composition differences between WT and *Mgll*^−/−^ mice at 6 weeks of age while the mice were on a standard chow diet (Figure 1A,C,D). However, already after 6 weeks of HFD feeding, *Mgll*^−/−^ mice gained significantly less weight than WT controls (Figure 1A), which was also evident by body composition analyses (from week 8, Figure 1C,D). *Mgll*^−/−^ mice exhibited reduced body fat mass and concomitantly increased lean mass as a percentage of body weight compared to WT controls. This difference persisted throughout the whole feeding period and was even more pronounced after 16 weeks of HFD feeding. To investigate whether differences in weight gain were a result of decreased food intake by *Mgll*^−/−^ mice fed HFD, we monitored weekly food intake from week 11 to week 16 but found no difference between *Mgll*^−/−^ and WT mice (Figure 1B). After 22 weeks on HFD, *Mgll*^−/−^ mice had improved glucose tolerance compared to WT mice as determined by an intraperitoneal glucose tolerance test (ipGTT; Figure 1E). These data support previous studies showing that *Mgll*^−/−^ mice are resistant to HFD-induced obesity and associated dysregulation of glucose homeostasis [28,30].

**Figure 2:**
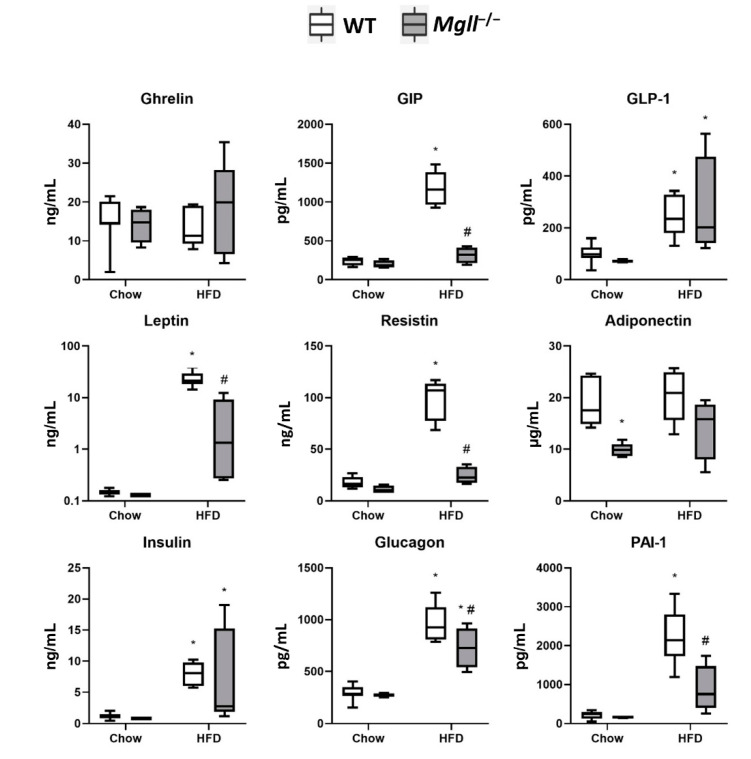
*Mgll*^−/−^ mice are resistant to HFD-induced changes in plasma peptide levels. Plasma peptide levels were assessed in WT and *Mgll*^−/−^ on chow or HFD for 22 weeks. Data is presented in box plots extending from the 25th to the 75th percentile with whiskers extending to the 10th and 90th percentile. * *p* < 0.05 vs. chow control, # *p* < 0.05 vs. WT control.

We then went on to assess the effects of *Mgll* deficiency on HFD-induced changes of circulating levels of peptides involved in the regulation of glucose homeostasis using a multiplexing immunoassay (Figure 2) in overnight fasted mice. After 22 weeks of HFD feeding, neither ghrelin nor adiponectin levels were altered in WT or *Mgll*^−/−^ mice compared to chow diet. However, adiponectin levels were lower in *Mgll*^−/−^ compared to WT mice when on chow diet, but this difference was lost due to a nonsignificant increase in *Mgll*^−/−^ adiponectin levels. Loss of *Mgll* did not alter HFD-induced increases in GLP-1 or insulin levels, which did not differ between genotypes on either chow or HFD diets. On the contrary, *Mgll*^−/−^ mice had significantly attenuated increases in glucagon levels in response to HFD as compared to WT mice, though glucagon did still increase significantly in the mutants. In the case of gut-derived GIP and adipose-derived leptin, resistin, and PAI-1, all of these factors showed no differences between *Mgll*^−/−^ and WT mice on a chow diet; however, the HFD-induced increases in all these factors were significantly reduced in *Mgll*^−/−^ mice, where the diet only resulted in nonsignificant trends. These data, taken together, show that *Mgll*^−/−^ mice are resistant to HFD-induced obesity, having decreased adiposity, increased insulin sensitivity, and significantly mitigated increases in many obesity- and diabetes-associated factors.

### 3.2. Mgll^−/−^ Mice Exhibit Alterations in the Gut Microbiome in Response to HFD Feeding.

**Figure 3:**
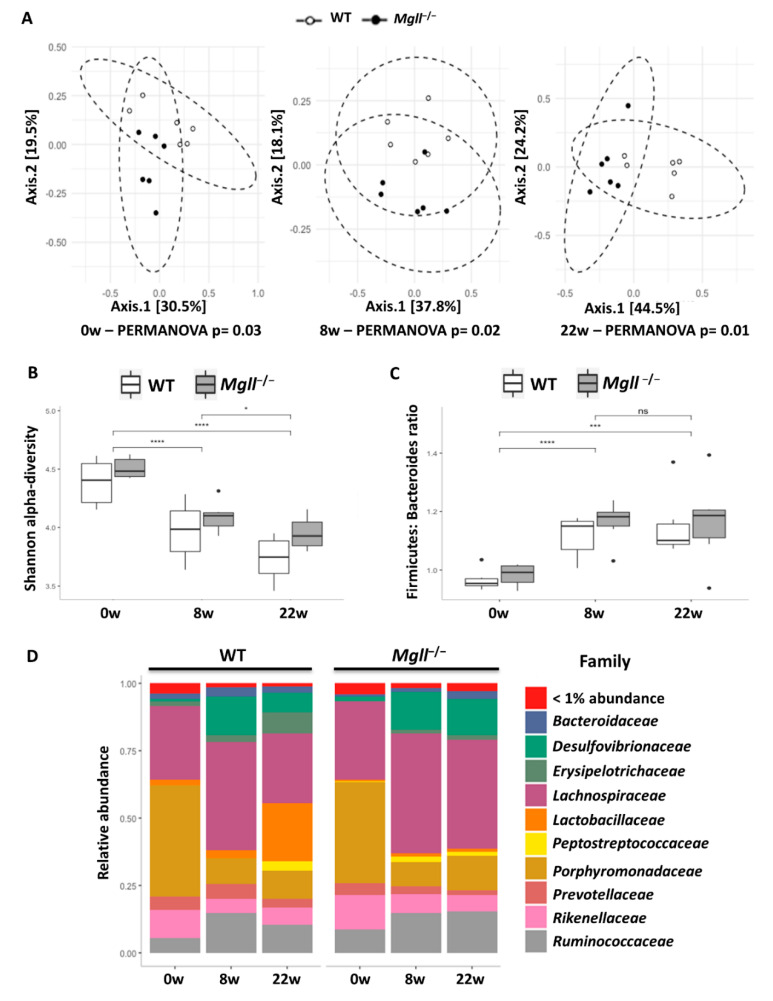
The microbiomes of WT and *Mgll^−/−^* mice differ under both chow and HFD feeding. (**A**) Principal coordinate analysis (PCoA)of microbiomes from WT and *Mgll^−/−^* mice on chow diets (0w; left), HFD feeding for 8 weeks (8w; middle) and 22 weeks (22w right). Permutational multivariate analysis of variance (PERMANOVA) p values are indicated for each time-point. (**B**,**C**) Shannon alpha-diversity index (**B**) evaluating gut microbiota richness and evenness and Firmicutes to Bacteroidetes ratio (**C**). Asterisks displayed above brackets represent Wilcoxon *p*-values * *p* < 0.05, *** *p* < 0.001, **** *p* < 0.0001, ns; not significant, between diets. No significant differences were obtained between genotypes. (**D**) Relative abundance according to genotype at family level.

**Figure 4:**
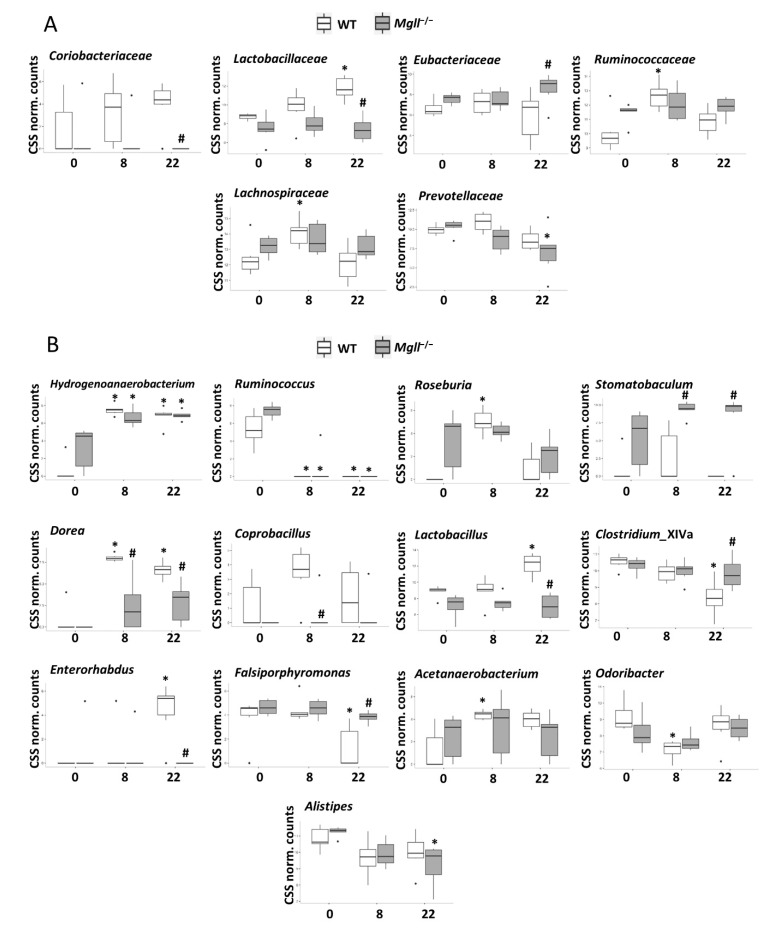
Bacterial taxa are differentially abundant within WT and *Mgll*^−/−^ mice on chow and HFD feeding. Fecal microbiomes were characterized in WT and *Mgll*^−/−^ mice under chow diet feeding (0) or HFD-feeding for 8 weeks (8) or 22 weeks (22) via 16rDNA sequencing. (**A**,**B**) Prevalence of bacterial families (**A**) and genera (**B**) showing differences between genotypes under the different diets or different responses to HFD feeding. * *p* < 0.05 vs. relevant chow control, # *p* < 0.05 vs. relevant WT control.

We next assessed the microbiomes of WT and *Mgll*^−/−^ mice on chow diet and in response to HFD feeding. Principal coordinate analysis (PCoA) and). Permutational multivariate analysis of variance (PERMANOVA) revealed distinct fecal microbiota composition between the genotypes at 6 weeks of age when fed chow diet (Figure 3A). However, Shannon alpha-diversity indexes (Figure 3B), describing both fecal microbial richness and evenness, and ratio of the *Firmicutes* to *Bacteroidetes* phyla (Figure 3C) were not different between the genotypes. Further, the most prevalent bacterial families in both genotypes were *Porphyromonadaceaea, Lachnospiraceae*, and *Rikenellaceae*, with no significant differences in abundance between genotypes (Figure 3D). However, some genotype differences in abundance of bacterial genera were observed during chow-feeding. Indeed, *Hydrogenoanaerobacterium* and *Roseburia* were only detected, and *Ruminococcus* was significantly higher, in *Mgll*^−/−^ mice, though these differences were eliminated after 8 weeks on the HFD (see below and Figure 4B). In response to the HFD, *Hydrogenoanaerobacterium* increased in abundance to the same level and remained stable, while *Ruminococcus* decreased to below detectable levels in both genotypes. *Roseburia*, however, increased significantly in abundance only in the WT mice after 8 weeks on the HFD and then returned to baseline after 22 weeks.

Gut microbiota are well known to be altered by HFD feeding in mice [41], as observed in both WT and *Mgll*^−/−^ mice (Figure 3). The differences between the genotypes observed on chow became more significant upon HFD feeding, with PERMANOVA *p*-values decreasing from 0.03 at 6 weeks on chow to 0.02 and 0.01 after 8 and 22 weeks of HFD, respectively (Figure 3A). As expected, in response to the HFD, Shannon alpha-diversity index was decreased in WT and *Mgll*^−/−^ mice to a similar extent (Figure 3B), suggesting that the number of different bacterial species and/or their evenness decreased in response to HFD feeding. Similarly, the often observed obesity-associated increase in *Firmicutes* to *Bacteroidetes* ratio was elevated during HFD-feeding in both genotypes (Figure 3C), suggesting that these diet-dependent alterations were not able to be overcome by the obesity-resistant phenotype of *Mgll*^−/−^ mice. Indeed, the abundances of most bacterial taxa were modified in response to the HFD irrespective of the genotype over the course of the protocol (16 of the 23 bacterial families and 36 of the 51 genera showed significant HFD-induced differences after 22 weeks) (Supplemental Table S2).

During HFD-feeding, *Lachnospiraceae*, *Desulfovibrionaceae*, and *Ruminococcaceae* gained in importance at the expense of *Porphyromonadaceaea*. When comparing bacterial abundances in *Mgll*^−/−^ and WT mice on HFD, we observed no differences at the family levels after 8 weeks; however, after 22 weeks, *Lactobacillaceae* and *Coriobacteriaceae* were significantly lower (or undetectable) and *Eubacteriaceae* were significantly higher in the mutants (Figure 4A). Interestingly, of these, an interaction between genotype and response to diet was only present for *Lactobacillaceae* abundance at 22 weeks, driven by a significant increase during HFD-feeding in WT mice over time, whereas it remained unaltered in *Mgll*^−/−^ mice. In fact, at the family level, a subset of microbiota responded to HFD-feeding in ways that were influenced by the *Mgll*^−/−^ genotype (Figure 4A). As with *Lactobacillaceae*, which increased in WT after 22 weeks on the HFD, *Ruminococcaceae* and *Lachnospiraceae* (*p* = 0.06) levels also increased in WT mice, but only at 8 weeks, while levels in *Mgll*^−/−^ mice remained constant. In contrast, *Prevotellaceae* remained unchanged in WT mice but were significantly decreased by HFD in *Mgll*^−/−^ mice.

Differences upon HFD feeding were also identified at the genera level (Figure 4B). While no HFD-induced changes were observed in WT mice for *Coprobacillus* or *Stomatobaculum*, these genera were less or more abundant in *Mgll*^−/−^ mice, respectively, only in the context of the HFD. *Dorea* however, were increased in both genotypes in response to the HFD, but much more dramatically in WT mice. In contrast, several genera were only responsive to the HFD in WT mice, with *Lactobacillus* and *Enterorhabdus* being increased, and *Clostridium*_XlVa and *Falsiporphyromonas* being decreased in WT mice, but unchanged in *Mgll*^−/−^ mice. Finally, some genera whose levels were not different between WT and *Mgll*^−/−^ mice under the HFD, responded differently to the HFD with respect to changes from baseline on the chow diet. For instance, *Roseburia* increased, and *Acetanaerobacterium* and *Odoribacter* decreased in WT mice, while *Alistipes* increased in *Mgll*^−/−^ mice at 8 and/or 22 weeks of HFD feeding. Taken together, these data indicate that the gut microbiomes of *Mgll*^−/−^ mice are significantly different from WT mice on chow diet and respond differently to chronic HFD feeding.

### 3.3. SCFA and Associations with Gut Microbiota

Given that SCFA levels are known to decrease in the feces of mice on an HFD, we measured the levels of these metabolites (acetate, propionate, butyrate, isobutyrate, valerate, and isovalerate) in the feces of *Mgll*^−/−^ and WT mice to better characterize the metabolic impact of *Mgll* deletion, as most bacterial taxa that were altered in *Mgll*^−/−^ mice are SCFA-producers. Of note, the HFD was significantly deprived of dietary fibers, precursors of SCFA. As expected, after 8 weeks on the HFD the levels of acetate, propionate, and butyrate were significantly decreased in WT mice and remained low at the end of the study, while the diet did not affect the levels of isobutyrate, valerate, or isovalerate (Supplemental Figure S1). Similar results were observed in the feces from *Mgll*^−/−^ mice, though there was a strong trend for decreased acetate and propionate levels (*p* < 0.1) after 8 weeks on the HFD. These differences became less pronounced after 22 weeks of feeding, suggesting that altered microbiomes of *Mgll*^−/−^ mice do not produce different levels of SCFAs under an HFD.

### 3.4. Associations of Gut Microbiota with Gut Peptides and Adipokines

**Figure 5:**
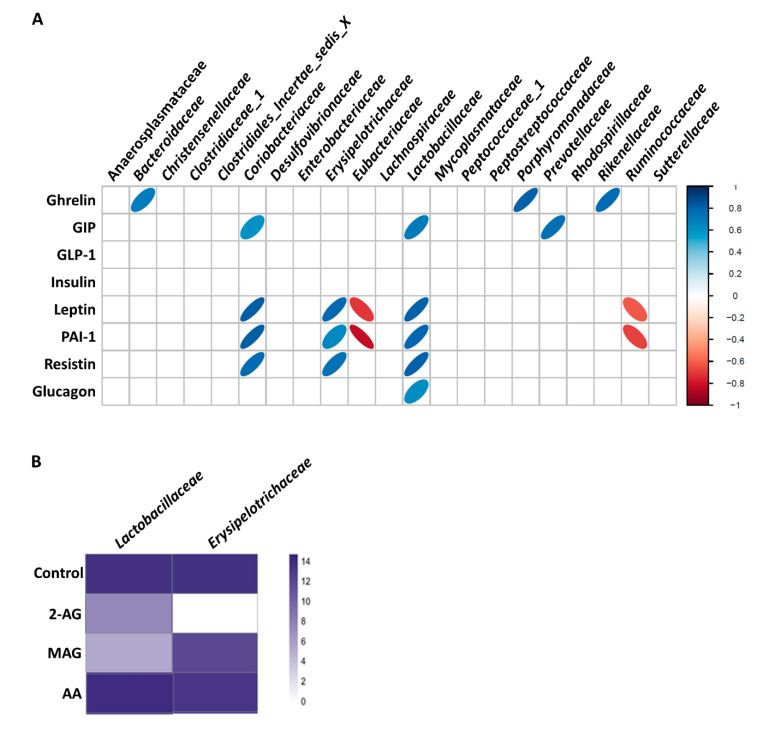
Bacterial families that are differentially abundant within feces of WT and *Mgll*^−/−^ mice correlate with plasma cytokine levels are sensitive to 2-AG and MAG in in vitro cultures. (**A**) Spearman rho coefficients were used to determine the relationship between bacterial families identified within feces of WT and *Mgll*^−/−^ mice after 22 weeks of HFD feeding. Ellipses drawn for correlations with *p* < 0.05. (**B**) Fecal material isolated from the intestine of a WT mouse was cultured in Thioglycolate Broth media (culture condition 12; see supplemental Table S1) supplemented without or with 2-AG (40 µM), MAGs (40 µM), or AA (40 µM) for 3 days. Resulting cultures were sequenced in order to characterize their microbial content and CSS-normalized counts are displayed for families replicating observations made in WT and *Mgll*^−/−^ mice.

In order to determine if the differences we observed in the levels of circulating factors relevant to diabetes (see above) in WT and *Mgll*^−/−^ mice after HFD feeding for 22 weeks correlated to alterations in microbial composition we performed a Spearman’s rank order correlation (Figure 5A). *Prevotellaceae* positively correlated with GIP, both of which decreased in *Mgll*^−/−^ mice under the HFD. Similarly, *Lactobacillaceae* and *Coriobacteriaceae*, that were also decreased in *Mgll*^−/−^ mice under HFD, showed strong positive correlations with GIP (rho = 0.71 and 0.60, respectively) as well as leptin (rho = 0.82 and 0.83, respectively), resistin (rho = 0.82 and 0.79, respectively), and PAI-1 (rho = 0.80 and 0.83, respectively), all of which were significantly decreased in the mutant mice as compared to WT. *Erysipelotrichaceae* was similarly positively correlated with leptin (rho = 0.79), resistin (rho = 0.74), and PAI-1 (rho = 0.69). While the abundance of this family was not altered between genotypes at any given time point, it exhibited a genotype effect, being decreased in *Mgll*^−/−^ mice when both chow and HFD data sets were pooled (*p* = 0.011). Conversely, *Ruminococcaceae*, which increased in WT but not *Mgll*^−/−^ mice in response to the HFD, and *Eubacteriaceae*, which was increased in *Mgll*^−/−^ mice vs. WT mice under the HFD, were negatively correlated with both leptin and PAI-1 levels. These data suggest that the alterations in gut peptide and adipokine levels induced by a HFD are related to HFD-induced changes in the gut microbiome, and that *Mgll*^−/−^ mouse resistance to HFD-induced and obesity-linked metabolic perturbations may be partly due to gut microbiome-associated alterations in these mediators.

### 3.5. 2-AG, MAGs, and Bacterial Growth

In an attempt to link the observed changes in the gut microbiome of *Mgll*^−/−^ mice with a direct effect of the increased levels of the endocannabinoid 2-AG and MAGs present in these mice, we performed a proof of principle experiment utilizing an in vitro culturomics approach. Fresh stool isolated from the intestine of a WT mouse was cultured in 18 different conditions (Supplemental Table S1) supplemented daily with 40 µM of either 2-AG, its fatty acid metabolite AA, or a purified MAG cocktail isolated from mouse intestines. After 3 days, cultures were subjected to 16S sequencing to characterize the bacterial composition of the individual cultures. We identified a culture condition (culture #12; supplemental Table S1) that led to changes in two of the five families identified as differently abundant between WT and *Mgll*^−/−^ mice when all experimental groups were pooled (ANOVA genotype *p*-values for *Lactobacillaceae*, *p* = 4.2^−6^; *Coriobacteriaceae*, *p* = 0.0029; *Eubacteriaceae*, *p* = 0.0095; *Erysipelotrichaceae*, *p* = 0.011, *Prevotellaceae*, *p* = 0.049). Specifically, *Lactobacillaceae* and *Erysipelotrichaceae*, both of which are decreased in *Mgll*^−/−^ mice, were found to have decreased abundance in media specifically supplemented with 2-AG and MAGs, but not AA, as compared to the control culture (Figure 5B). This proof of principle experiment suggests that the decreased abundance of these families in *Mgll*^−/−^ mice may be, at least in part, a direct result of increased MAG and/or 2-AG levels within the intestines of *Mgll*^−/−^ mice.

## 4. Discussion

MGLL plays a major role in the degradation of the endocannabinoid 2-AG, related bioactive long-chain MAGs, and MAGs in general, resulting in the formation of free fatty acids from these molecules, including AA serving as a precursor for other bioactive metabolites. We showed here that mice lacking this enzyme exhibit a different gut microbiome profile as compared to WT mice on the same genetic background, both under a normal chow diet and, particularly, following an obesogenic and insulin resistance inducing HFD.

We confirmed that *Mgll*^−/−^ mice accumulate less fat and become less glucose intolerant than WT mice following HFD. No weight or body composition differences were observed on the chow diet at baseline, unlike those reported by Douglass et al. in association with increased activity/energy expenditure; however, it should be noted that in that study the mice were assessed at 8 weeks, and not at 6 weeks as we have, which may explain the differences in our observations [28]. *Mgll*^−/−^ mice on the HFD also presented an altered profile of gut and adipose tissue-derived hormones and mediators. As expected, they had muted HFD-induced increases in the blood levels of the proinflammatory and insulin desensitizing adipokines, leptin and resistin. While we have not measured leptin sensitivity in our mice and are unaware of any data showing that *Mgll*^−/−^ mice do not become leptin resistant after an extended period of time on an HFD, unlike WT mice [42,43], the lower levels of leptin we observed in HFD-fed *Mgll*^−/−^ mice suggest this. Given that decreasing dietary fat content in HFD-induced obese mice quickly restores leptin sensitivity and glucose metabolism in mice [42], we postulate that the reduced fat absorption in *Mgll*^−/−^ mice [20] could potentially contribute to conserved leptin sensitivity in these mice under HFD, a possibility that deserves investigation in future work. Additionally, the increased insulin sensitivity of *Mgll*^−/−^ mice on an HFD, which has been previously shown by Taschler et al. [20], may be linked to lower resistin levels in these mice under HFD, given that resistin can induce insulin resistance [44], independent of the lack of any observed changes in adiponectin or ghrelin levels.

*Mgll*^−/−^ mice also exhibited a lower increase in the circulating concentrations of PAI-1, which has been involved in atherogenic inflammation and plaque stability [45,46]. This finding might explain in part the previous report of a propensity to develop more stable atherosclerotic plaques of *Mgll*^−/−^ as compared to WT mice, following an atherogenic diet. Interestingly, patients with coronary artery disease have an increase in the order *Lactobacillales* [47], which we found here to be significantly lower in *Mgll*^−/−^ mice on HFD (*p* = 0.003 at 22 weeks; data not shown), suggesting that alterations in the gut microbiome of *Mgll*^−/−^ mice may also contribute to the resistance of these mice to the development of atherosclerosis and supporting the proposition that the gut microbiome-eCBome axis may be relevant to cardiovascular health [20].

Indeed, obesity per se or as a risk factor for atherosclerosis due to excessive abdominal fat and insulin resistance, has been associated to perturbations of the gut microbiome (gut dysbiosis). Here, interesting differences in gut microbiome profiles according to the *Mgll*^−/−^ genotype were observed, suggesting that the establishment of HFD-induced gut dysbiosis may be attenuated in *Mgll*^−/−^ mice, thus partly explaining their resistance to diet-induced obesity, insulin resistance, and atherogenesis. Accordingly, WT mice seemed to be more susceptible to microbiome modification as they exhibited more variations in response to HFD. For example, the *Prevotellaceae* family, reported to be increased in obese individuals and reduced via gastric bypass surgery [48] and associated to HFD-induced obesity in mice [49], was decreased during the experiment in *Mgll*^−/−^ mice. *Coriobacteriaceae* was more abundant in WT mice following 22 weeks of HFD, which is consistent with the findings of others that this family correlates positively with both body weight and proinflammatory cytokine levels [50]. This family is also suspected of being detrimental to cholesterol metabolism, being positively correlated with non-HDL plasma concentrations and reduced with a dietary intervention that improved cholesterol homeostasis in hamsters [51]. Interestingly, *Mgll*^−/−^ mice have reduced cholesterol levels when fed a HFD as compared to controls [28] (though others have reported only trends in a western diet) [40], which one could speculate may be related in part to decreased *Coriobacteriaceae* levels. However, the idea that *Coriobacteriaceae* may regulate cholesterol metabolism is still controversial, as there is also evidence that improved cholesterol metabolism related to increased biliary cholesterol excretion results in deceases in *Coriobacteriaceae* as well as *Erysipelotrichaceae* levels [51]. The decreases in these two families in *Mgll*^−/−^ mice may be the result of the host altered cholesterol metabolism phenotype given that these mice exhibit increased cholesterol elimination via the biliary pathway [30]. Wilkins et al. recently identified some bacterial taxa as potential markers of dysbiosis for their depletion from healthy gut community or enrichment in diseases [52]. At the genus level, the often obesity associated *Dorea* was among these dysbiosis markers and was observed here as being more abundant in WT mice and to be further increased by HFD. Similarly, the presence of *Lactobacillus*—and the *Lactobacillaceae* family and *Lactobacillus* genus—were more elevated during HFD-feeding in WT than *Mgll*^−/−^ mice, a modification that is often observed in the establishment of HFD-induced obesity [53,54]. *Eubacteriaceae* is another family that has been positively associated with leanness vs. obesity following HFD [55], and we found it here to be more abundant in *Mgll*^−/−^ mice following 22 weeks of HFD (G). The *Clostridium_XIVa* genus, which is normally enriched in the gut communities of healthy and lean individuals genus [55], was decreased by the HFD in WT mice, while *Mgll*^−/−^ mice seemed to be protected from such a potentially deleterious alteration. It should be noted that changes in gut microbiome in response to HFD occur even in the absence of obesity [41], which may be in part due to the direct interaction of the microbiome with increased levels of fat within the feces. This is not insignificant, especially in the case of *Mgll*^−/−^ mice, which have decreased intestinal fat absorption [40,56], which would in theory expose gut microbes to even more fat in the gut lumen than in WT mice and therefore differentially impact upon microbial composition.

Many of the bacteria that were differentially abundant between WT and *Mgll*^−/−^ mice are known to produce SCFAs. As expected, HFD feeding reduced fecal SCFA levels; however, there was no difference between WT and *Mgll*^−/−^ on either diet and no fecal SCFAs have been found to associate with healthful metabolic parameters, such as insulin sensitivity [57], while fecal levels have been correlated with obesity and cardiovascular risk factors [58]. It is possible that *Mgll*^−/−^ mice have increased absorption of SCFAs and thus increased levels within blood, an interesting possibility that merits future examination.

Interestingly, previous studies in mice showed that pharmacological blockade of CB1 activity with the CB1 antagonist rimonabant or chronic administration of the CB1 agonist Δ^9^-tetrahydrocannabidiol (THC), a condition shown to lead to CB1 desensitization similar to that observed in *Mgll*^−/−^ mice [20,31,59], also demonstrated gut microbiota variations. The CB1 antagonist induced depletion of *Firmicutes* (namely *Lachnospiraceae* and *Erysipelotrichaceae*) and elevation of *Akkermansia muciniphila* during diet-induced obesity [10]. Similar elevations of *Akkermansia muciniphila* were observed with chronic THC administration but with an increase of *Firmicutes* [12], though the expected elevation of the *Firmicutes* to *Bacteroidetes* ratio during HFD was prevented by THC. We reported here that, similar to CB1 pharmacological antagonism, in *Mgll*^−/−^ mice the levels of *Erysipelotrichaceae* are decreased, suggesting that this family may be responsive to changes in CB1 activity given its downregulation in knockout mice [20,56]. Regarding elevations of the *Firmicutes* to *Bacteroidetes* ratio during HFD-feeding, we found that the WT and *Mgll*^−/−^ genotypes responded similarly, whereas *Akkermansia muciniphila* was unfortunately not detected in our samples.

The positive metabolic effects of THC reported in mice appear translate to humans, and studies using several large cohorts of individuals have correlated cannabis use to decreased BMI and protective effects against diabetes (reviewed in [4]). Interestingly chronic cannabis use is also associated with alterations in bacterial populations, including a decreased abundance of the genus *Dorea*, which is associated with obesity [60,61,62]. The reduced levels of *Dorea* within *Mgll*^−/−^ mice on a HFD that we observed is similar to that observed in cannabis users, given that these mice have desensitized CB1 similar to that seemingly occurring in chronic cannabis use [62]. It would be interesting to speculate that this genus responds to decreased CB1 signaling, perhaps within the gut or elsewhere within the body.

The impact of the modulation of endocannabinoids and eCBome mediators on the gut microbial community was previously investigated also in another type of mutant mice, i.e., those with targeted adipocyte or intestinal epithelial cell deletion of the *N*-acylethanolamine (NAE)-synthesizing enzyme, NAPE-PLD, which were accompanied by disruption of different gut microbiota taxa depending on the cell targeted [7,8]. Interestingly, some of these taxa were also affected by the *Mgll*^−/−^ genotype. At the family and genus levels, adipocyte-*Napepld* or global *Mgll* knockouts affected the abundance of *Coriobacteriaceae* (elevated in WT) and *Erysipelotrichaceae*; and the *Allobaculum, Bacteroides*, *Clostridium*, and *Lactobacillus* genera (elevated in WT), respectively. *Napepld^−/−^* knockout always reduced the tissue levels of NAEs (except for anandamide in the adipose tissue), without affecting those of MAGs (except for an increase of 2-AG in the small intestine) [7]. Both NAEs and MAGs target various non-CB1 (and eCBome family member) receptors, including the hot chili pepper receptor, TRPV1, and the incretin-stimulating receptor GPR119. The positive metabolic effects of dietary capsaicin, the active chili pepper component, and prototypical TRPV1 agonist, have also been associated with alterations in the gut microbiome [9,63,64]. Like capsaicin, NAEs and MAGs activate and subsequently desensitize TRPV1 [33], and ablation or capsaicin-induced stimulation of this channel result in a decrease or increase, respectively, in *Lactobacillus* genera in mice [63,65], i.e., the same or the opposite of what we found in *Mgll*^−/−^ mice. Although the changes previously observed with capsaicin may have been due to direct stimulatory effect on *Lactobacillus* metabolic activity as has been observed in vitro [66], it is tempting to speculate that some of the common alterations found in the gut microbiome of *Mgll*^−/−^ or cell-specific *Napepld^−/−^* null mice are due in part to TRPV1 desensitization or inhibition, due to excessive MAG or reduced NAE tissue levels, respectively. By contrast, the fact that we did not see any difference between *Mgll*^−/−^ and WT mice in GLP-1 release, the first and most important effect of activation of GPR119 [67], suggests that this latter receptor is instead unlikely to be involved in any of the phenotypic differences observed here between the two genotypes. On the other hand, plasma GIP levels, another response associated with GPR119 activation, were not elevated in *Mgll*^−/−^ mice following a HFD, as they were in WT mice, in agreement with previous findings of a negative role of this peptide in obesity and/or insulin resistance [68]. In fact, the plasma levels of endocrine mediators were found here to strongly correlate with the relative abundance of some gut bacterial families in a manner (positively or negatively) that was generally in agreement with the reciprocal proposed protective or exacerbating roles of these peptides in obesity and diabetes and with the aforementioned proposed metabolic function of the microbial families therewith correlated. The family *Eubacteriaceae*, which was increased in *Mgll*^−/−^ mice, negatively correlated with plasmatic mediators increased by the HFD, while the families *Coriobacteriaceae* and *Lactobacillaceae*, which were decreased in *Mgll*^−/−^ mice, positively correlated with them. This is in line with studies from humans, which have found an increased abundance of *Coriobacteriaceae* and *Lactobacillaceae* in association with obesity [69]. Furthermore, in mice, *Eubacteriaceae* abundance was increased by probiotic- and exercise/dietary fiber supplementation-mediated weight loss and improvement in insulin sensitivity, and was associated with increased lean mass [70,71,72], which we find is increased in *Mgll*^−/−^ mice under an HFD

As with capsaicin, the possibility exists that 2-AG is able to directly affect specific bacteria in some manner that will modulate their abundance within the gut. While this has not been demonstrated directly for 2-AG, the other canonical AA-derived endocannabinoid and CB1 agonist, AEA has been found to have antimicrobial activity against *Staphylococcus aureus* [73] as did various fatty acid containing MAGs [74]. As aforementioned, *Mgll*^−/−^ mice not only have higher 2-AG levels within their intestines [56], but also increased levels of 16:0- (palmitic acid-containing), and 18:2- (linoleic acid-containing) MAGs. Therefore in order to test if the alterations in bacterial composition observed in *Mgll*^−/−^ mice could be the result of a direct action of 2-AG or MAGs, we designed a proof of principle culturomics-based experiment in which 18 different culture conditions supplemented without or with 2-AG, total MAGs isolated from intestines or free AA were used to culture bacteria obtained from the intestinal tract (duodenum to colon) of a mouse. Sequencing of the resulting cultures identified a culture condition that was permissive for two bacterial families (*Lactobacillaceae* and *Erysipelotrichaceae*), which were reduced in *Mgll*^−/−^ mice. Within this culture condition both of these families were reduced in abundance specifically by 2-AG and MAG, but not AA, supplementation. Interestingly while *Lactobacillaceae* abundance appeared to be similarly affected by 2-AG and MAGs, *Erysipelotrichaceae* appeared to be more strongly affected by 2-AG. These data suggest that, at least for a subset of bacteria, their abundance in *Mgll*^−/−^ mice may be directly influenced by increased 2-AG and or MAG levels within the intestinal tract, and not due to physiological changes within the mice resulting from a lack of MGLL activity and subsequent overstimulation of MAG receptors in host cells. Accordingly, a study published during revision of this manuscript showed that 2-AG can directly inhibit the virulence of enteric pathogens via a non-CB1-mediated, and QseC-mediated, mechanism [75]

## 5. Conclusions

The bidirectional interaction between the gut microbiome and the eCBome reported recently opens the potential of further understanding how these systems regulate metabolic health. In this study we examined the effect of the deletion of the eCBome metabolic enzyme monoglyceride lipase (MGLL) in mice (*Mgll*^−/−^), in which the tissue levels of the endocannabinoid (eCB) 2-AG and some of its MAG congeners are increased, on the gut microbiome. These mice are resistant to diet-induced obesity and metabolic disturbances, however, the involvement of the gut microbiome with relation to this phenotype has not been examined. Our results show that *Mgll*^−/−^ mice have an altered gut microbiome that may contribute to their obesity-resistant phenotype. Furthermore, some microbial families that we identified as being differentially abundant in *Mgll*^−/−^ mice were similarity affected in cultures supplemented with 2-AG and MAGs, providing further evidence for the presence of a gut microbiome-eCBome axis that is relevant to the regulation of metabolic health.

## Supplementary Materials

The following are available online at https://www.mdpi.com/2073-4409/9/12/2705/s1, lemental Figure S1. Fecal SCFA levels are not different between WT and *Mgll*^−/−^ mice. Supplemental Table S1: Culture conditions used in the targeted culturomics, Supplemental Table S2. Changes in abundances of bacterial taxa according to Diet, Genotype, and Genotype*Diet over the course of the protocol.
