# Supplementary material for: Mgll Knockout Mouse Resistance to Diet-Induced Dysmetabolism Is Associated with Altered Gut Microbiota
Source: Cells. 2020 Dec 17;9(12):2705. doi: 10.3390/cells9122705 (PMC7765900; doi:10.3390/cells9122705)
Supplement: Supplementary file 1 [file cells-09-02705-s001.pdf]

Supplemental Materials

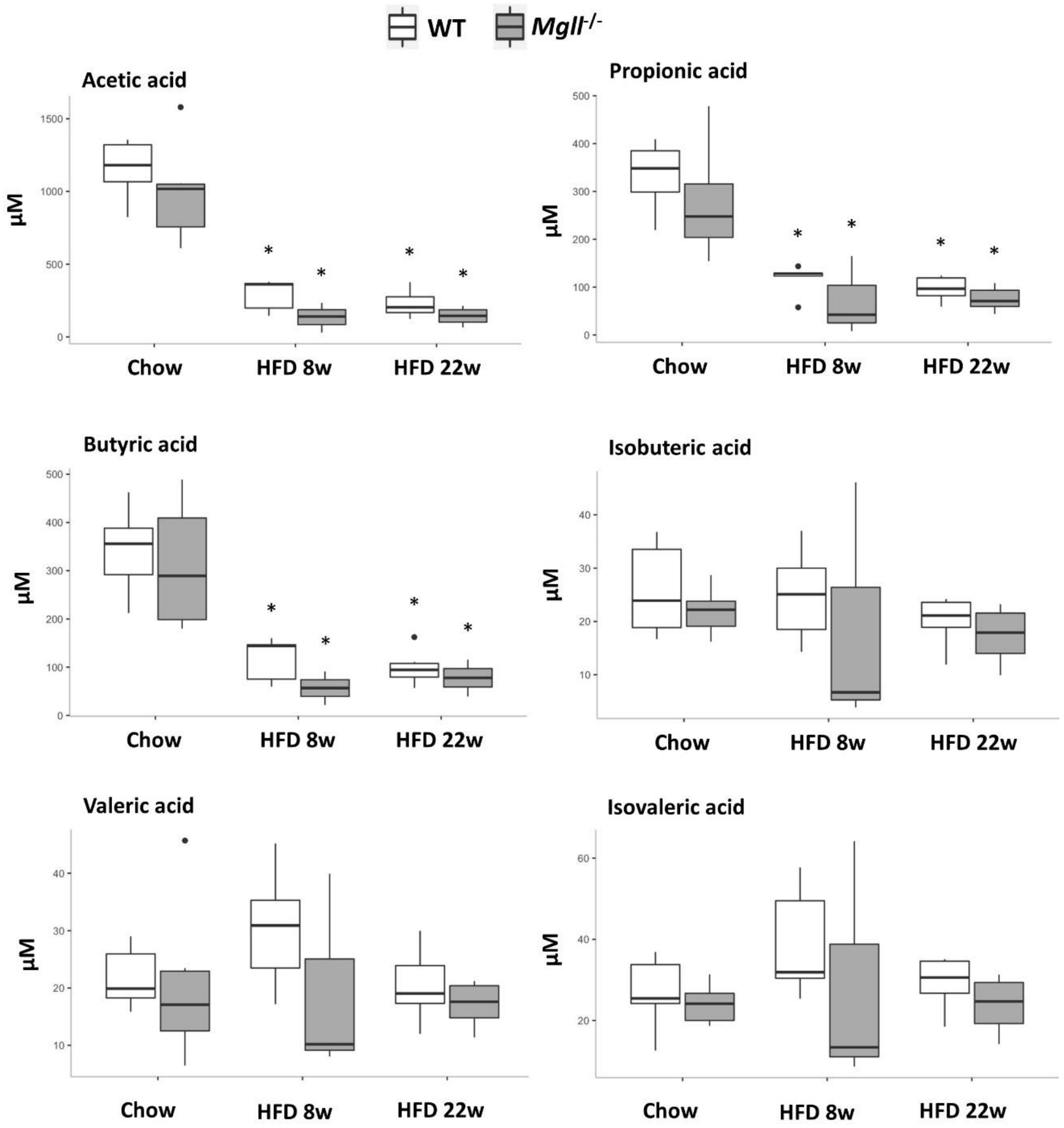

Supplemental Figure S1. Faecal SCFA levels are not different between WT and *Mgll*$^{-/-}$ mice. SCFA levels were measured in faecal samples from WT and *Mgll*$^{-/-}$ mice on chow diets and after 8 and 22 weeks of HFD feeding. * $p < 0.05$ vs. relevant chow control .

Supplemental Table S1. Culture conditions used in the targeted culturomics.

Culture Conditions	Media Composition
**1.** Preincubation in BACTEC Lytic Anaerobic medium with 5% sheep blood and 5% of rumen fluid, anaerobic condition at 37 °C	BACTEC Lytic Anaerobic medium (BD, Franklin Lakes, New Jersey, USA); Sheep blood (Fisher scientific, Hampton, New Hampshire, USA); Rumen fluid (Centre de recherche en sciences animales de Deschambault)
**2.** Preincubation in BACTEC Lytic Anaerobic medium with 5% of rumen fluid, anaerobic condition at 37 °C	BACTEC Lytic Anaerobic medium (BD, Franklin Lakes, New Jersey, USA); Rumen fluid (Centre de recherche en sciences animales de Deschambault, Quebec, Canada)

**3.** Preincubation in BACTEC Lytic Anaerobic medium with 5% sheep blood, anaerobic condition at 37 °C	BACTEC Lytic Anaerobic medium (BD, Franklin Lakes, New Jersey, USA); Sheep blood (Fisher scientific, Hampton, New Hampshire, USA)
**4.** Preincubation in BACTEC Lytic Anaerobic medium after thermic shock at 80°C/20mn, anaerobic condition at 37°C	BACTEC Lytic Anaerobic medium (BD, Franklin Lakes, New Jersey, USA); Sheep blood (Fisher scientific, Hampton, New Hampshire, USA); Rumen fluid (Centre de recherche en sciences animales de Deschambault, Quebec, Canada)
**5.** Preincubation in BACTEC Lytic Anaerobic medium with stool filtered at 5µm, anaerobic condition at 37°C	BACTEC Lytic Anaerobic medium (BD, Franklin Lakes, New Jersey, USA); Sheep blood
**6.** Preincubation in Chopped Meat Medium Carbohydrate (CMC), anaerobic condition at 37°C	Chopped Meat Medium Carbohydrate (Morgan Hill, California, USA)
**7.** Preincubation in Chopped Meat Medium (CM), anaerobic condition at 37°C	Chopped Meat Medium (Morgan Hill, California, USA)
**8.** Preincubation in Yeast Casitone Fatty Acids broth with Carbohydrates -YCFAC Broth, anaerobic condition at 37°C	Yeast Casitone Fatty Acids broth with Carbohydrates - YCFAC (Morgan Hill, California, USA)
**9.** Preincubation in MTGE Broth anaerobic condition at 37°C	MTGE Broth (Morgan Hill, California, USA)
**10.** Preincubation in PYG Broth after thermic shock at 80°C/20mn, anaerobic condition at 37°C	Peptone Yeast Extract Broth with Glucose-PYG broth
**11.** Preincubation in Brain Hart Infusion (BHI) Broth with stool filtered at 5µml, anaerobic condition at 37°C	Brain Hart Infusion (Anaerobe systems, Morgan Hill, California, USA)
**12.** Preincubation in Thioglycolate Broth, anaerobic condition at 37°C	Thioglycolate Broth (Anaerobe systems, Morgan Hill, California, USA)
**13.** Preincubation in BACTEC Lytic Anaerobic medium with 5% sheep blood and 5% of rumen fluid, aerobic condition at 37°C	BACTEC Lytic Anaerobic medium (BD, Franklin Lakes, New Jersey, USA); Sheep blood (Fisher scientific, Hampton, New Hampshire, USA); Rumen fluid (Centre de recherche en sciences animales de Deschambault)
**14.** Preincubation in BACTEC Lytic Anaerobic medium with rumen fluid, aerobic condition at 37°C	BACTEC Lytic Anaerobic medium (BD, Franklin Lakes, New Jersey, USA); Rumen fluid (Centre de recherche en sciences animales de Deschambault
**15.** Preincubation in BACTEC Lytic Anaerobic medium with 5% sheep blood, aerobic condition at 37°C	BACTEC Lytic Anaerobic medium (BD, Franklin Lakes, New Jersey, USA); Sheep blood (Fisher scientific, Hampton, New Hampshire, USA)
**16.** Preincubation in Columbia broth, anaerobic condition at 37°C	Columbia broth (Sheep blood (Fisher scientific, Hampton, New Hampshire, USA)
**17.** Preincubation in BACTEC Lytic Anaerobic medium with stool filtered at 5µm, aerobic condition 37°C	BACTEC Lytic Anaerobic medium (BD, Franklin Lakes, New Jersey, USA)
**18.** Preincubation in Brain Hart Infusion (BHI) Broth, aerobic condition at 37°C	Brain Hart Infusion (Anaerobe systems, Morgan Hill, California, USA)

Supplemental Table S2. Changes in abundances of bacterial taxa according to Diet, Genotype and Genotype*Diet over the course of the protocol.

Bacteria family	p-value		
	**Diet**	**Genotype**	**Geno*Diet**

Anaeroplasmataceae	2.6e-05	0.071	ns
Bacteroidaceae	ns	ns	ns
Bdellovibrionaceae	0.00017	ns	ns
Christensenellaceae	0.058	ns	ns
*Clostridiaceae*_1	7.3e-09	ns	ns
*Clostridiales*_Incertae_Sedis_XIII	0.011	ns	ns
Coriobacteriaceae	ns	0.0029	ns
Desulfovibrionaceae	6.6e-09	0.099	0.091
Enterobacteriaceae	ns	ns	ns
Erysipelotrichaceae	ns	0.011	ns
Eubacteriaceae	ns	0.0095	ns
Lachnospiraceae	0.0082	ns	ns
Lactobacillaceae	0.036	4.2e-06	0.015
Mycoplasmataceae	0.031	ns	ns
*Peptococcaceae*_1	5.1e-07	ns	ns
Peptostreptococcaceae	0.0029	ns	ns
Porphyromonadaceae	6.3e-06	ns	ns
Prevotellaceae	0.0032	0.049	ns
Rhodospirillaceae	ns	ns	ns
Rikenellaceae	0.023	ns	ns
Ruminococcaceae	0.0017	0.084	0.047
Sutterellaceae	8.2e-12	ns	ns
No match	6.1e-05	ns	ns
**Bacterial genera**		**p-value**	
	**Diet**	**Genotype**	**Geno*Diet**
Acetanaerobacterium	0.031	ns	ns
Acetatifactor	1.8e-12	ns	0.068
Acetitomaculum	0.046	ns	ns
Aestuariispira	ns	ns	ns
Alistipes	0.0014	ns	ns
Allobaculum	ns	0.021	ns
Alloprevotella	ns	0.038	ns
Anaeroplasma	2.4e-05	0.1	ns
Anaerostipes	0.0071	ns	ns
Anaerotruncus	0.001	ns	ns
Anaerovorax	0.0069	0.095	ns
Bacteroides	ns	0.06	ns
Barnesiella	1.5e-14	ns	ns
Butyricicoccus	9.8e-06	ns	ns
Christensenella	ns	ns	ns
*Clostridium*_III	0.0026	ns	ns
*Clostridium*_IV	2.8e-05	ns	ns
*Clostridium*_XlVa	0.00044	0.079	0.019
*Clostridium*_XlVb	ns	ns	ns
Coprobacillus	0.1	0.002	ns
Coprobacter	0.0051	ns	ns
Coprococcus	ns	0.012	ns
Desulfovibrio	ns	ns	ns
Dorea	1.8e-07	4.9e-06	0.0086
Eisenbergiella	1.1e-11	ns	0.023
Enterorhabdus	0.04	0.039	0.0016

Escherichia	ns	ns	ns
Eubacterium	ns	0.02	ns
Falsiporphyromonas	0.001	0.0055	0.056
Flavonifractor	1.3e-06	ns	ns
Gemmiger	1e-04	ns	ns
Hydrogenoanaerobacterium	5.3e-11	ns	0.009
Intestinimonas	0.013	ns	ns
Lactobacillus	0.012	6.1e-07	0.0033
Marvinbryantia	0.072	ns	ns
Odoribacter	0.0016	ns	ns
Oscillibacter	7.4e-12	ns	ns
Parabacteroides	ns	ns	ns
Parasutterella	1.1e-11	ns	ns
Peptococcus	3.2e-08	ns	ns
Prevotella	7.1e-11	ns	ns
Pseudoflavonifractor	4.6e-05	ns	ns
Robinsoniella	0.0077	ns	ns
Romboutsia	0.0024	ns	ns
Roseburia	1.9e-05	0.022	0.008
Ruminococcus	5.9e-15	0.018	0.097
Ruminococcus2	0.0012	ns	0.078
Stomatobaculum	0.073	5.1e-07	ns
Ureaplasma	0.058	ns	ns
Vampirovibrio	0.00011	ns	ns
No match	8.3e-05	ns	0.071
